# Metabolite Alterations in Adults With Schizophrenia, First Degree Relatives, and Healthy Controls: A Multi-Region 7T MRS Study

**DOI:** 10.3389/fpsyt.2021.656459

**Published:** 2021-05-19

**Authors:** S. Andrea Wijtenburg, Min Wang, Stephanie A. Korenic, Shuo Chen, Peter B. Barker, Laura M. Rowland

**Affiliations:** ^1^Department of Psychiatry, Maryland Psychiatric Research Center, University of Maryland School of Medicine, Baltimore, MD, United States; ^2^The Russell H. Morgan Department of Radiology and Radiological Science, Johns Hopkins University School of Medicine, Baltimore, MD, United States; ^3^FM Kirby Research Center for Functional Brain Imaging, Kennedy Krieger Institute, Baltimore, MD, United States

**Keywords:** 7T MRS, schizophrenia, first degree relatives, brain, metabolism, glutamate, glutamine, GABA

## Abstract

Proton magnetic resonance spectroscopy (MRS) studies in schizophrenia have shown altered GABAergic, glutamatergic, and bioenergetic pathways, but if these abnormalities are brain region or illness-stage specific is largely unknown. MRS at 7T MR enables reliable quantification of multiple metabolites, including GABA, glutamate (Glu) and glutamine (Gln), from multiple brain regions within the time constraints of a clinical examination. In this study, GABA, Glu, Gln, the ratio Gln/Glu, and lactate (Lac) were quantified using 7T MRS in five brain regions in adults with schizophrenia (*N* = 40), first-degree relatives (*N* = 11), and healthy controls (*N* = 38). Metabolites were analyzed for differences between groups, as well as between subjects with schizophrenia with either short (<5 years, *N* = 19 or long (>5 years, *N* = 21) illness duration. For analyses between the three groups, there were significant glutamatergic and GABAergic differences observed in the anterior cingulate, centrum semiovale, and dorsolateral prefrontal cortex. There were also significant relationships between anterior cingulate cortex, centrum semiovale, and dorsolateral prefrontal cortex and cognitive measures. There were also significant glutamatergic, GABAergic, and lactate differences between subjects with long and short illness duration in the anterior cingulate, centrum semiovale, dorsolateral prefrontal cortex, and hippocampus. Finally, negative symptom severity ratings were significantly correlated with both anterior cingulate and centrum semiovale metabolite levels. In summary, 7T MRS shows multi-region differences in GABAergic and glutamatergic metabolites between subjects with schizophrenia, first-degree relatives and healthy controls, suggesting relatively diffuse involvement that evolves with illness duration. Unmedicated first-degree relatives share some of the same metabolic characteristics as patients with a diagnosis of schizophrenia, suggesting that these differences may reflect a genetic vulnerability and are not solely due to the effects of antipsychotic interventions.

## Introduction

Recent proton magnetic resonance spectroscopy (MRS) studies in schizophrenia (SZ) have shown altered GABAergic (1–3), glutamatergic (4–7), and more recently, bioenergetic pathways (8) across multiple brain regions and illness durations. Often these studies quantify metabolites from one or two brain regions within a single session, and many have been performed at the standard field strength of 3T. An advantage of higher field strength magnets, such as 7T, is the increased signal to noise ratio (SNR) and improved spectral separation compared to lower field strengths (9–11). This can be used to acquire high quality MRS data in a shorter period of time, or alternatively to improve the measurement precision in similar periods of time. Several 7T MRS studies have reported good reproducibility in difficult to quantify metabolites like glutamate, glutamine, and GABA (12–14). Most previous 7T studies in schizophrenia have focused only on the anterior cingulate (7, 8, 15, 16) while others have expanded to acquisitions from two or three brain regions within a session (3, 17, 18). A recent study used 7T MRS to examine group differences in first episode patients (2 years or less illness duration) and healthy controls in five brain regions (19); however, there has not yet been a study that examined multiple brain regions thought to be involved in the pathophysiology of schizophrenia which also considers illness phase, or genetic risk for schizophrenia by investigating first-degree relatives (FDRs).

In this study, GABA, glutamate (Glu), glutamine (Gln), lactate, and the ratio of Gln/Glu were quantified from five brain regions [anterior cingulate cortex (ACC), dorsolateral prefrontal cortex (DLPFC), centrum semiovale (CSO), thalamus (Thal), and hippocampus (HP)] in each of the three groups: participants with schizophrenia (SZ), first-degree relatives (FDR), and healthy controls (HC). Glu and GABA, the primary excitatory and inhibitory neurotransmitters in the human brain, were included because animal and post-mortem studies have implicated them in the pathophysiology of SZ (20–22). Since 80% of glutamine is involved in glutamatergic neurotransmission (23), is quantifiable at 7T, and shown to be altered in previous SZ studies (24, 25), it was also included as a metabolite of interest. Total glutamate levels measured by MRS cannot differentiate glutamate involved in neurotransmission, GABA synthesis, protein synthesis, etc.; however, the ratio of Gln/Glu has been suggested as an index for glutamatergic neurotransmission (26, 27), so is also reported here. Lactate was included due to its integral role in energy metabolism, recent post-mortem SZ studies showing increased lactate in several brain regions including the DLPFC and hippocampus (28), and our prior work in a smaller sample size that showed elevated lactate levels in adults with SZ were related to reduced cognitive function and functional capacity (8). The five regions of interest (ACC, CSO, DLPFC, HP, and Thal) were chosen due to their implicated role in the pathophysiology of SZ in pre-clinical, post-mortem, and neuroimaging studies. The ACC and hippocampus have been extensively studied in SZ across the illness duration (19, 26, 29–34) so were included. Studies focused on the thalamus, a key node in many functional circuits, showed reduced thalamic volume in adults with SZ (35) and altered glutamatergic metabolites in clinical high risk for psychosis and chronic SZ participants (30, 36, 37). The CSO was included because it is a predominantly white matter region, and white matter is increasingly implicated as altered in SZ (38, 39). The DLPFC was included because reduced GAD67 mRNA expression, leading to reduce GABA synthesis in parvalbumin-expressing subpopulation of GABA neurons has been found in SZ and as noted above elevated Lac levels (20). First-degree relatives were chosen because they may share common, albeit sub-threshold psychopathology, traits as SZ patients. This group is genetically (40, 41), physiologically (42), cognitively (43, 44), and neurochemically (17, 45, 46) similar to adults with SZ, but without exposure to antipsychotic medications. In addition, within the SZ group, metabolite differences between short (<5 years illness duration) and long (>5 years illness duration) were examined as previous studies have suggested an aging effect in SZ (2, 4). Further, MRS studies of SZ suggest that Glu and Gln may change across the course of the illness with elevated glutamatergic metabolite levels being reported in clinical high risk for psychosis (30, 47) and first-episode (19, 48) populations. In contrast, lower glutamatergic metabolite levels have been reported in chronic SZ populations (31, 37). To our knowledge, this will be the first study to examine glutamatergic metabolites in multiple brain regions in SZ participants with short and long illness durations.

## Methods

This study was approved by the University of Maryland Baltimore and the Johns Hopkins University School of Medicine Institutional Review Boards, and all participants provided written informed consent prior to study enrollment. Forty adults with SZ [*N* = 19 with short illness duration (<5 years) and *N* = 21 with long illness duration (>5 years)], 38 healthy controls (HC), and 11 FDRs participated in this study. Some data from 29 HC and 27 SZ were previously reported (8). Participant demographics are shown in Table 1. The Brief Psychiatric Rating Scale (BPRS) (49) for assessment of positive symptoms and general psychopathology and the Brief Negative Symptom Scale (BNSS) (50) were administered to each participant with SZ. The BPRS evaluates the participant's positive symptoms such as suspiciousness, grandiosity, unusual thought content, and hallucinations. The BNSS contains specific subscales that evaluate the participant's negative symptoms such as anhedonia, asociality, avolition, blunted affect, and alogia. In addition, the Structured Clinical Interview for DSM-IV-TR (SCID), the UCSD Performance-Based Skills Assessment (UPSA) (51), Level of Functioning test (LOF) (52), and the MATRICS battery (53, 54) were administered to all participants. The UPSA assesses a participant's functional capacity in areas of household chores, communication, finance, transportation, and planning recreational activities. The LOF assesses the participant's physical functioning, personal care skills, interpersonal relationships, social acceptability, activities of community living, and work skills. The MATRICS battery evaluates the participant's processing speed, attention, verbal and non-verbal working memory, verbal learning, visual learning, reasoning, and social cognition. For all groups, inclusion criteria were: (1) age range: 18–55 years old; (2) no lifetime substance dependence or abuse in the last 6 months; (3) no contraindication for 7T MRI scanning; (4) not pregnant or nursing; (5) no major medical or neurological illness. HC were included if he/she did not have psychiatric illness, while adults with SZ had a diagnosis of SZ or schizoaffective. FDRs have a first degree relative with SZ, but are not related to anyone in the SZ group. Also, FDRs did not have a DSM-IV psychosis diagnosis or current substance abuse or dependence. 10 out of 11 participants were not taking psychotropic medications.

**Table 1:** Participant demographics.

	**SZ**	**FDR**	**HC**
Mean Age (years ± stdev)	34.2 ± 12.4	37.2 ± 12.7	30.5 ± 10.5
Gender (M/F)	23/17	2/9	19/19
Mean Education Level (years ± stdev)^**^	12.9 ± 1.6	14.5 ± 1.9	14.4 ± 1.8
Smoking Status (Yes/No)	11/29	1/10	8/30
Mean Illness Duration (years ± stdev)	12.4 ± 11.9	N/A	N/A
Cognitive Variables			
Matrics Composite Score (mean ± stdev)^**^	28.5 ± 14.0	46.5 ± 13.0	44.1 ± 8.9
UPSA Score (mean ± stdev)^**^	87.4 ± 19.5	101.5 ± 13.3	101.4 ± 9.5
LOF Score (mean ± stdev)^**^	23.2 ± 5.2	34.7 ± 2.0	33.6 ± 3.6
Symptom Ratings			
BPRS total score (mean ± stdev)	38.1 ± 8.1	N/A	N/A
BPRS positive score (mean ± stdev)	7.9 ± 3.6	N/A	N/A
BPRS negative score (mean ± stdev)	6.8 ± 2.3	N/A	N/A
BNSS score (mean ± stdev)	16.3 ± 10.5	N/A	N/A
Chlorpromazine Equivalents (mean ± stdev)	336.7 ± 329.0		

** *statistically significant (p < 0.05), FDR, first degree relative; HC, healthy control; stdev, standard deviation; SZ, schizophrenia*.

### Imaging Protocol

**Figure 1:**
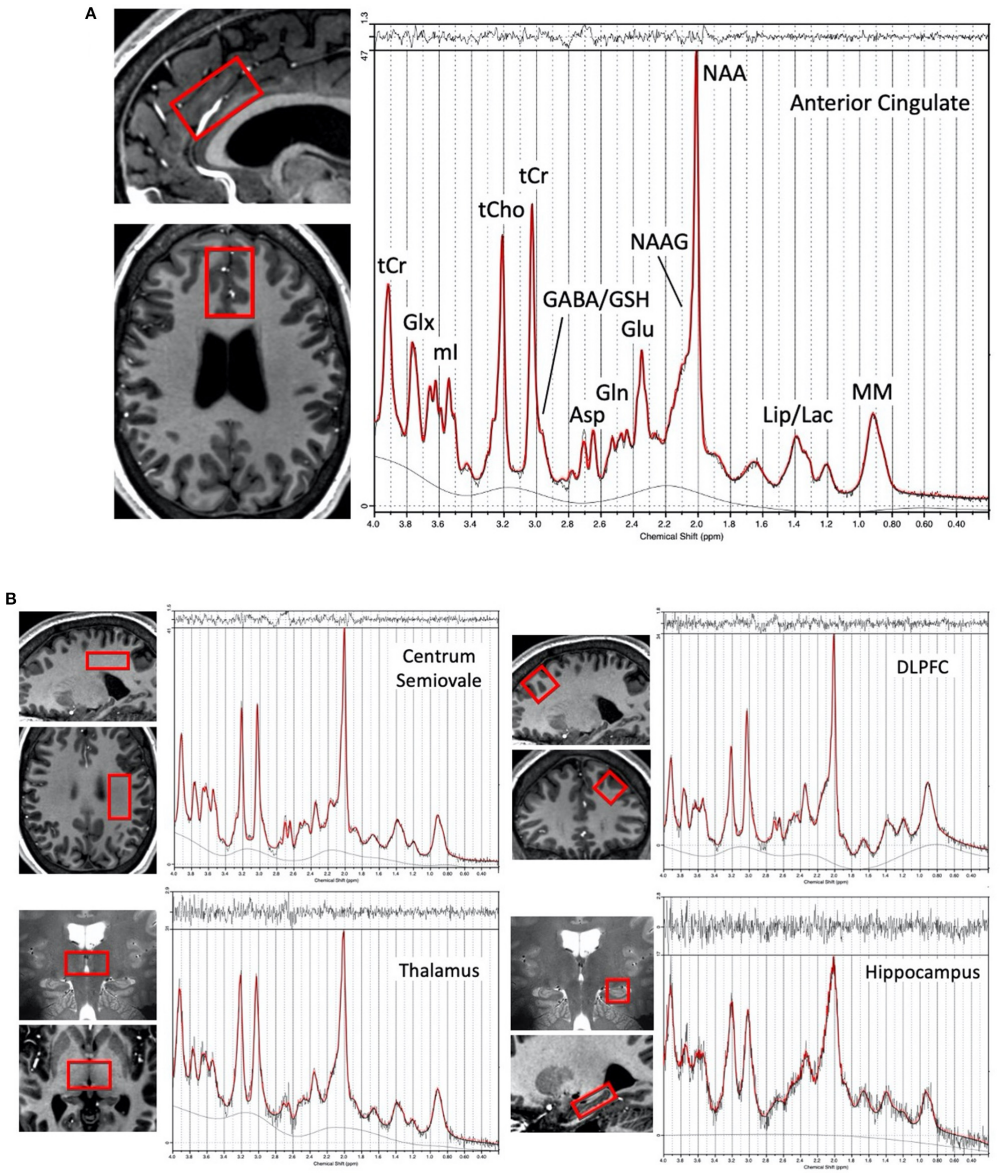
**(A)** Representative voxel location chosen for the anterior cingulate cortex region in one subject (female, 28 yrs. old) and proton spectrum (LCModel output in red) with peak assignments indicated, **(B)** Representative voxel locations (overlaid in two planes on T_1_ or T_2_-weighted images) from the left centrum semiovale, left dorsolateral prefrontal cortex, bilateral thalamus, and left hippocampus with a corresponding representative spectrum from each region.

A 7T Philips Achieva scanner (Best, the Netherlands) equipped with a dual-transmit and 32-channel receive head coil (Nova Medical, Wilmington, MA) was used to scan each participant. Using an isotropic 0.8 mm T_1_-weighted MPRAGE sequence for guidance, spectroscopic voxels were prescribed in ACC, left CSO, left DLFPC, left hippocampus, and bilateral thalamus (Figure 1). Voxel sizes were 12 (3 × 2 × 2) cm^3^ in the ACC, 12 (4 × 2 × 1.5) cm^3^ in the CSO, 10 (2.5 × 2 × 2) cm^3^ in the DLPFC, ~7.9 (3.5 × 1.5 × 1.5) cm^3^ in the hippocampus, and 9 (2 × 3 × 1.5) cm^3^ in the thalamus. Spectroscopic data were acquired using a STEAM sequence with TR/TM/TE = 3,000/33/14 ms, VAPOR (55) water suppression, and 128 excitations for all regions. A spectrum without water suppression (two excitations) was also acquired for phase and eddy current correction, and use as a quantitation reference (scan time 6 min 30 s per region for both suppressed and non-suppressed acquisitions). Prior to acquisition, shimming was applied up to 2nd order using a projection based method (56), and a localized power optimization performed (57) on the region of interest. High-resolution (0.5 × 0.5 × 1.0 mm) multi-slice coronal T_2_-weighted images were also recorded.

Spectra were analyzed using the ‘LCModel' software package version 6.3 (58). A basis set was created using the ‘VESPA' program (59), and chemical shifts and coupling constants from (60) for the following metabolites: alanine (Ala), aspartate (Asp), creatine (Cr), γ-aminobutyric acid (GABA), glutamate (Glu), glutamine (Gln), glutathione (GSH), glycerophosphocholine (GPC), glycine (Gly), lactate (Lac), *myo*-inositol (mI), N-acetylaspartate (NAA), N-acetylaspartylglutamate (NAAG), phosphocholine (PCh), phosphocreatine (PCr), phosphorylethanolamine (PE), *scyllo*-inositol (sI), serine (Ser), and taurine (Tau). In the LCModel, a fit range of 0.6–4.0 ppm was used and the baseline spline control parameter (“dkntmn”) set to 0.2 (58). All metabolites except lactate were corrected for the proportion of cerebrospinal fluid (CSF) within the MRS voxel, using [X]_corrected_ = [X]/(1-f_CSF_) where [X] is the metabolite concentration [expressed in in institutional units (i.u.)] as output by LCModel, and f_CSF_ is the fraction of CSF within the voxel (8). f_CSF_, as well as the fractions of the gray and white matter within the voxel, were calculated by segmentation of the anatomical T_1_-weighted images using the “SPM12” program. Lactate was not corrected for the proportion of CSF because this metabolite can be quantified from the CSF using MRS (61, 62). No metabolite relaxation time corrections were performed.

Data were excluded from further analyses if the full-width half maximum (FWHM) > 0.1 ppm for all regions and SNR ≤ 10 for ACC, CSO, DLPFC or SNR ≤ 5 for hippocampus and thalamus (values reported by LCModel). There following datasets were excluded from further analyses: two from the AC (1 HC and 1 SZ), one from the CSO (1 FDR), two from DLPFC (2 SZ), 14 from the hippocampus (7 HC, 6 SZ, and 1 FDR), and none from the thalamus. Metabolites were included in statistical analyses if at least 75% of the possible datasets met the CRLB criteria listed of CRLBs ≤ 20% (Asp, Cr, GABA, Glu, GPC, GSH, mI, NAA, PCh, PCr, PE, sI, Tau, tCho, tNAA, tCr, Glx, mI+Gly) or CRLBs ≤ 30% (Ala, Gln, Gly, Lac, NAAG, Ser). In terms of the main metabolites of interest, Lac analyses were only conducted in the AC and CSO while GABA, Glu, Gln, and Gln/Glu analyses were conducted in all five regions.

### Statistics

Due to non-normality, non-parametric tests (Kruskal-Wallis, Mann-Whitney or chi-square) were utilized to assess group differences between HC, SZ, and FDR for demographic variables, voxel composition, spectral quality, and metabolite levels. Based on the stated hypotheses, group differences were examined in each region for GABA, Glu, Gln, Gln/Glu, and Lac with significance set to *p* < 0.05. False discovery rate correction for multiple comparisons was performed for each metabolite across the five regions (*p* < 0.05). In addition, non-parametric tests (Mann-Whitney) were utilized to assess group metabolite level differences between patients with short (<5 years illness duration) or long (>5 years illness duration) illness durations. Similar to the three group analyses, group differences were assessed for GABA, Glu, Gln, Lac, and Gln/Glu ratio with significance set to *p* < 0.05, and false discovery rate correction for multiple comparisons was performed across the five regions (*p* < 0.05). To examine whether the differences between illness duration groups were a function of illness length or an age effect, non-parametric tests (Mann-Whitney) were conducted to assess metabolite differences between the short illness duration SZ group and an age-matched (within 1 year) subset of HC as well as non-parametric ANCOVAs with age as a covariate to assess metabolite differences between the short and long illness duration groups. Significance was set to *p* < 0.05.

For the three groups, Spearman's rho correlations were performed for cognitive and function variables (MATRICS total score, UPSA, and LOF) and MRS metabolite levels. Only metabolites that were significantly different between groups were used in the correlation analyses with significance set to *p* < 0.05. Within the SZ groups, Spearman's rho correlations were performed between symptom severity (BPRS total, BPRS positive, and BNSS) and metabolites that were statistically significant between groups with significance set to *p* < 0.05.

## Results

### Participant Demographics

Participant demographics are given in Table 1. Adults with SZ, HC, and FDR did not differ in terms of age (H = 298, *p* = 0.23), gender (X^2^ = 3.9, *p* = 0.14), and smoking status (*X*^2^ = 2.0, *p* = 0.37). There was a significant difference in education level (H = 11.25, *p* = 0.004) such that adults with SZ had less years of formal education than HC. The FDR's education level was not significantly different from HC or SZ. Adults with SZ had lower total LOF score (H = 57.1, *p* < 0.001), MATRICS total score (H = 25.5, *p* < 0.001), and UPSA total score (H = 13.33, *p* = 0.001) compared to HC and FDR.

### Group Differences in Metabolites

**Figure 2:**
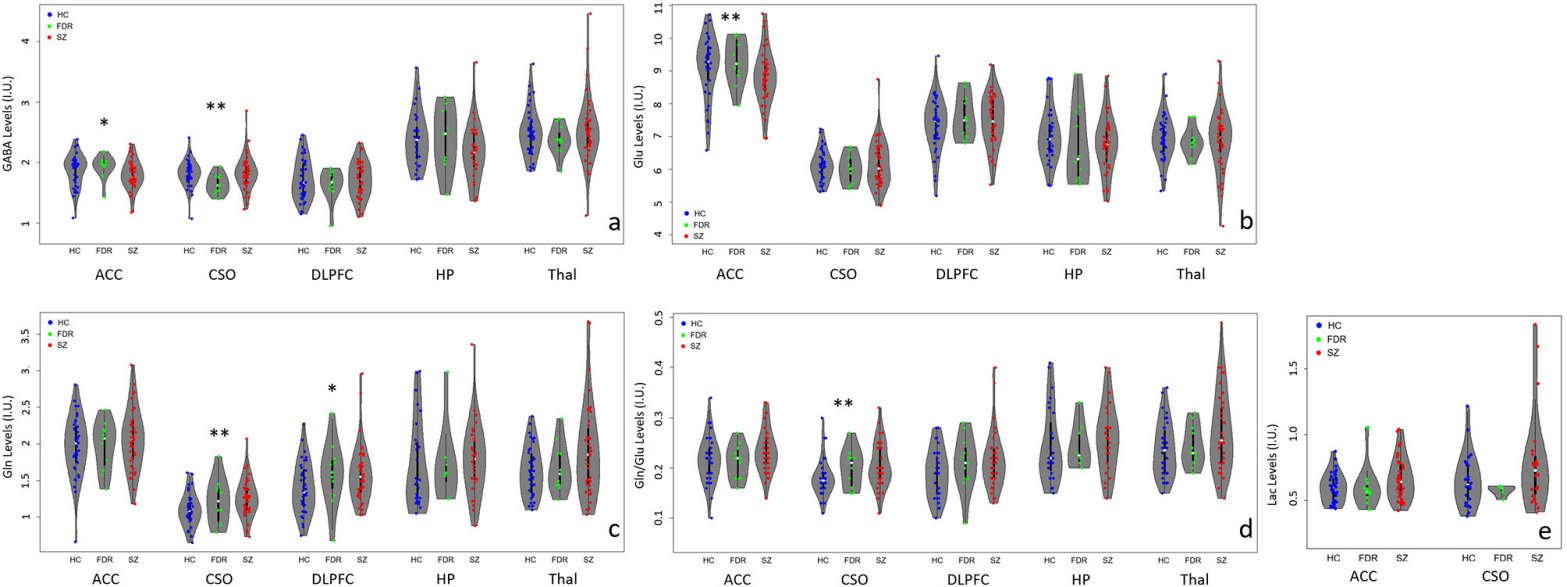
Violin plots with individual data points highlighting metabolite differences between adults with SZ (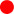), first-degree relatives (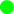), and healthy controls (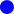) in the anterior cingulate cortex (ACC), left centrum semiovale (CSO), left dorsolateral prefrontal cortex (DLPFC), left hippocampus (HP), and bilateral thalamus (Thal). **(a)** GABA levels differed significantly between groups in the CSO (**p < 0.05) and at trend level (*p < 0.1) in the ACC. ACC GABA was significantly lower in the SZ compared to the HC and FDR groups. CSO GABA in the FDR group was trend level lower than the SZ or HC groups. **(b)** Glu levels differed significantly between groups in the ACC only such that adults with SZ had lower Glu levels than HC and FDR. **(c)** CSO Gln levels were significantly higher in the SZ group compared to HC and FDR while DLPFC Gln levels in the HC group was lower at trend level than the SZ and FDR groups. **(d)** CSO Gln/Glu ratio was significantly higher in the SZ group compared to the other two groups. **(e)** There were no significant Lac findings.

Means and standard deviations for the main metabolites for each group are summarized in Figure 2 and Supplementary Table 1. The supplement (Supplementary Table 2) information regarding voxel GM, WM, and CSF percentage differences and quality metrics FWHM and SNR as reported by the LCModel for the three groups. Of the five regions investigated, the SNR was the highest in the ACC followed by the CSO, DLPFC, thalamus, and hippocampus. Similarly, the narrowest FWHM was measured from the ACC, followed by the CSO and DLPFC, then the thalamus and hippocampus. For completeness, mean, standard deviations, N, and statistical significance for other quantifiable metabolites e.g., NAA, mI, tCr, tCho, etc. that may be of interest are summarized in the supplement (Supplementary Table 5).

In the ACC, Glu levels were significantly different between groups (*H* = 6.4, *p* = 0.04) such that adults with SZ had lower Glu levels than HC and FDR.There were no significant group differences for GABA, Gln, Gln/Glu ratio, or Lac. After applying correction for multiple comparisons, there were no significant group differences for the other quantifiable metabolites (*p*'s = 0.168–0.933).

In the CSO, GABA, Gln, and Gln/Glu ratio were significantly different between groups with (*H* = 6.8, *p* = 0.034, *H* = 7.4, *p* = 0.025, and *H* = 9.2, *p* = 0.010), respectively. Gln and Gln/Glu were significantly higher in the SZ group compared to the HC group. The FDR group's Gln and Gln/Glu levels fell in between the other two groups but were not significantly different. GABA levels were similar between SZ and HC but were significantly lower in the FDR group. After applying correction for multiple comparisons, there were no significant group differences for the other quantifiable metabolites (*p*'s = 0.05–0.98).

In the DLPFC, hippocampus, and thalamus, there were no significant differences for GABA, Glu, Gln, and Gln/Glu ratio among the three groups. In addition, there were no other significant group differences for the remaining quantifiable metabolites.

### Correlations Between Metabolites and Cognitive Measures

**Figure 3:**
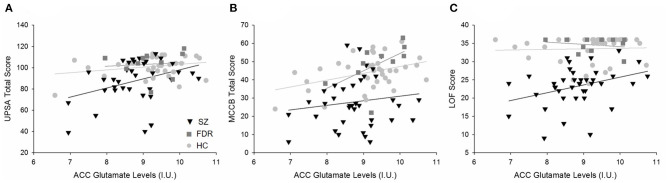
Regression plots of the significant relationships between **(A)** ACC Glu levels and UPSA total scores, **(B)** ACC Glu and MATRICS total scores, and **(C)** ACC Glu levels and LOF scores. **(A)** Separating by diagnostic group revealed a trend level relationship in only the SZ group (H) and not the HC (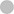) or FDR (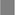). **(B)** ACC Glu levels were significantly correlated to MATRICS total score across diagnostic groups. Further analyses revealed a significant relationship in the FDR group only. **(C)** Similarly, ACC Glu levels were significantly correlated to LOF scores across diagnostic groups and in the SZ group. There were no significant relationships between HC and FDR. Similarly, ACC Glu levels were significantly correlated to MATRICS total score across diagnostic groups. Further analyses revealed a significant relationship in the FDR group only.

In the ACC, Glu was the only metabolite significantly different between groups. ACC Glu was strongly correlated to UPSA (rho = 0.321, *p* = 0.004) across groups (Figure 3A). Separating by diagnostic group revealed a trend correlation only in the SZ group (rho = 0.329, *p* = 0.054), and not in the HC (*p* = 0.511) or FDR (*p* = 0.374) groups. ACC Glu was also strongly correlated with LOF (rho = 0.364, *p* = 0.001) across groups and within the SZ group (rho = 0.360, *p* = 0.024) (Figure 3B). There were no significant correlations in HC (*p* = 0.229) or FDR (*p* = 0.728) groups. Finally, ACC Glu was also significantly correlated with MATRICS total score (rho = 0.383, *p* < 0.001) across groups, within the FDR group (rho = 0.693, *p* = 0.026), but not within the HC (*p* = 0.132) or SZ (*p* = 0.524) groups (Figure 3C). After correction for multiple comparisons, all three combined group correlations between Glu with UPSA, LOF, and MATRICS total score remained significant.

For the CSO, Gln and Gln/Glu were significantly correlated with LOF but not with other measures. Gln/Glu was correlated with LOF across groups (rho = −0301, *p* = 0.005) and within the SZ group (rho = −0.362, *p* = 0.023) but not HC (*p* = 0.671) or FDR (*p* = 0.377). Similarly, Gln was correlated with LOF (rho = −0.229, *p* = 0.036) across groups, but did not remain significant at the diagnostic group level (*p*'s = 0.076–0.96). GABA did not correlate with any measure. After correcting for multiple comparisons, none of these correlations remained significant.

### Early vs. Later Illness Duration

#### Participant Demographics

Adults with SZ with shorter (<5 years) and longer (>5 years) illness durations differed significantly with age (*U* = 21, *p* < 0.001) such that the shorter illness duration group was younger than the longer illness duration group (Table 2). The two illness groups did not differ on education level (*p* = 0.49), gender (*p* = 0.18), smoking status (*p* = 0.39), and CPZ equivalents (*p* = 0.73). There were no significant group differences for LOF (*p* = 0.18), UPSA (*p* = 0.07) MATRICS total score (*p* = 0.09), BPRS total (*p* = 0.71), BPRS positive (*p* = 0.16), and BNSS (*p* = 0.32) scores.

**Table 2:** Participant demographics for short and long illness duration.

	**Short**	**Long**
Mean Age (years ± stdev)^**^	24.3 ± 3.9	43.1 ± 11.0
Gender (M/F)	13/6	10/11
Mean Education Level (years ± stdev)	13.1 ± 1.0	12.8 ± 2.0
Smoking Status (Yes/No)	4/19	7/21
Mean Illness Duration (years ± stdev)	2.7 ± 1.7	21.2 ± 10.3
Cognitive Variables		
Matrics Composite Score (mean ± stdev)	32.7 ± 13.4	24.6 ± 13.7
UPSA Score (mean ± stdev)	94.6 ± 13.2	80.7 ± 22.3
LOF Score (mean ± stdev)	24.7.2 ± 4.4	21.9 ± 5.7
Symptom Ratings		
BPRS total score (mean ± stdev)	37.2 ± 6.8	39.0 ± 9.2
BPRS positive score (mean ± stdev)	7.1 ± 3.3	8.6 ± 3.7
BPRS negative score (mean ± stdev)	6.7 ± 2.3	6.9 ± 2.3
BNSS score (mean ± stdev)	14.1 ± 9.2	18.2 ± 11.3
Chlorpromazine Equivalents (mean ± stdev)	290.9 ± 286.0	372.8 ± 362.8

** *statistically significant (p < 0.05)*.

#### Metabolite Differences Between Short vs. Long Illness Duration

**Figure 4:**
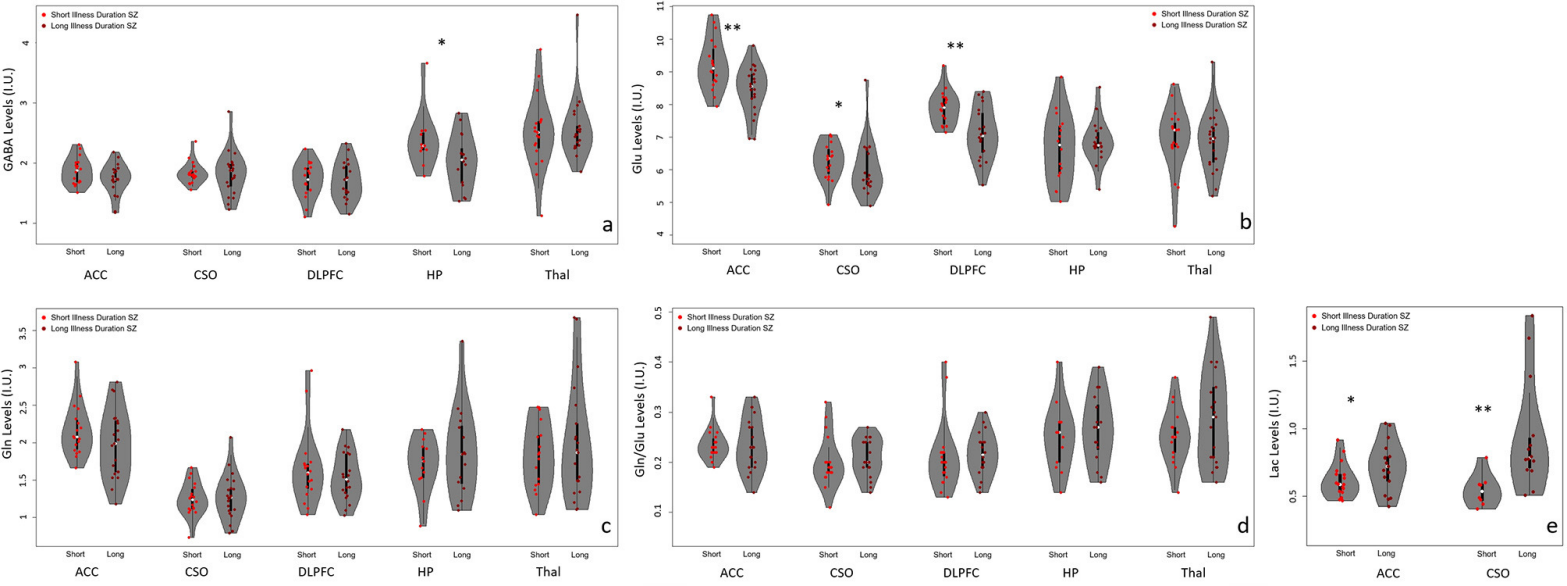
Violin plots with individual data points summarizing the significant (**p < 0.05) and trend level (*p < 0.1) differences between adults with SZ with short (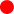) and long illness (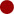) durations in the anterior cingulate cortex (ACC), left centrum semiovale (CSO), left dorsolateral prefrontal cortex (DLPFC), left hippocampus (HP), and bilateral thalamus (Thal). **(a)** There was a trend level difference in GABA in the HP such that GABA was higher in the short illness duration group compared to the long illness duration group. **(b)** Glu levels were significantly higher in the short illness duration group vs. the long illness duration group in both the ACC and DLPFC. A similar relationship, but at trend level, was observed in the CSO. **(c,d)** There were no significant relationships between groups across all regions for Gln and Gln/Glu. **(e)** For Lac, there was a significant difference in the CSO such that the long illness duration group had higher Lac levels compared to the short illness duration group. A similar relationship was observed in the ACC but at trend level.

Means and standard deviations for the main metabolite levels for the two groups are summarized in Figure 4 and Supplementary Table 3, and the supplement contains information regarding the voxel GM, WM, and CSF percentage differences and quality metrics (FWHM and SNR as reported by LCModel) for the two groups (Supplementary Table 2). In addition, results from other quantifiable metabolites e.g., NAA, mI, tCr, tCho, etc. are summarized in the supplement (Supplementary Table 6).

In the ACC, there were significant group differences for Glu (*U* = 286, *p* = 0.006), but not for GABA, Gln, or Gln/Glu. Glu levels were higher in the early illness duration group vs. the later illness duration group. Lac levels between groups were trend level different (*U* = 96, *p* = 0.06) such that Lac levels were higher in the longer illness duration vs. the shorter illness duration group. After correcting for multiple comparisons for the other metabolites, Glx was also different between groups (*U* = 284, *p* = 0.007) such that higher Glx was observed in the short compared to the longer illness duration group. No other quantifiable metabolites were significantly different between groups.

In the CSO, Lac levels were significantly different between groups (*U* = 13, *p* = 0.003) such that the longer illness duration group had higher levels of Lac compared to the shorter illness duration group. However, the sample size for each group, particularly the short illness duration group, for the Lac analyses was much smaller than the sample size for the other metabolites so this finding should be interpreted with caution. There were no Gln, Glx, Gln/Glu, or GABA differences between groups. No other quantifiable metabolites were significantly different between groups.

In the DLPFC, there was a significant difference between groups for Glu (*U* = 285, *p* = 0.002) such that the short illness duration group had higher Glu levels than the long illness duration group. There were no significant group differences for Gln, Gln/Glu, or GABA. After correction for multiple comparisons for the other quantifiable metabolites, Glx (*U* = 279, *p* = 0.003) was the only metabolite with significant differences between groups. Glx was higher in the shorter illness duration group.

In the hippocampus, there here were no significant group differences for GABA, Glu, Gln, or Gln/Glu ratio. After correcting for multiple comparisons for the other quantifiable metabolites, there were no significant differences between groups.

In the thalamus, there were no significant group differences for GABA, Glu, Gln, or Gln/Glu ratio. After correcting for multiple comparisons for the other quantifiable metabolites, mI (U = 96, *p* = 0.008) and mI+Gly (U = 92, *p* = 0.006) were significantly different such that mI and mI+Gly were significantly higher in the long illness duration group vs. the short illness duration group.

Detailed results of the group difference analyses between age-matched short illness duration group and HC group are in the supplement (Supplementary Text and Supplementary Table 7). CSO Lac and ACC Glu were the only two metabolites that were different (significantly for Lac at *p* = 0.037 and trend level for Glu at *p* = 0.083) between the short illness duration and HC analyses. Detailed results regarding the illness duration group difference analysis that co-varied for age are in the Supplementary Text. Results from the non-parametric ANCOVA revealed no significant differences between the short and long illness duration groups for any metabolites after controlling for age; however, given the highly correlated nature of age and illness duration, these results should be interpreted with caution.

#### Correlations Between Metabolites and Clinical Measures

ACC Glu and Glx were significantly different between the three groups (SZ, FDR, HC) or the two patient groups and therefore waere included in correlation analysis. ACC Glu was trend level related to BNSS (rho = −0.290, *p* = 0.072), and not significantly related to BPRS total or BPRS positive symptom score. Separating by illness duration revealed a significant relationship between ACC Glu and BNSS (rho = −0.445, *p* = 0.043) for the long illness duration group only. ACC Glx was not significantly related to the symptom ratings in the SZ group as a whole (*p*'s > 0.129) or when broken down by short or long illness duration groups (all *p*'s > 0.146). None of the relationships presented survived multiple comparison correction.

For the CSO, Gln, Gln/Glu, GABA, and Lac were significantly different between the three groups (SZ, FDR, HC) or the two patient groups and therefore were included in the correlation analysis. Gln was significantly associated with BPRS total score (rho = 0.363, *p* = 0.023) across groups, and a similar relationship was present in the long (rho = 0.433, *p* = 0.050) both not short (*p* = 0.335) and illness duration groups. Gln/Glu was significantly associated with BNSS (rho = 0.362, *p* = 0.023) across groups, and a similar trend relationship was present in the short illness duration group (rho = 0.468, *p* = 0.05) but not the long (*p* = 0.479) illness duration group. The other metabolites were not significantly associated with any of the symptom ratings (all *p*'s > 0.07). Similar to ACC, these correlations did not survive correction for multiple comparisons.

DLPFC Glu, and Glx, as well as thalamus mI and mI+Gly were significantly l different between the three groups (SZ, FDR, HC) or the two patient groups and therefore were included in the correlation analysis. There were no significant relationships between symptom rating and these metabolites for the SZ group as a whole (all *p*'s > 0.095), but there was a relationship between thalamus mI+Gly and BPRS total symptoms in the long illness duration group only (rho = 0.472, *p* = 0.031). This did not survive correction for multiple comparisons.

## Discussion

This study examined metabolite differences in five brain regions in adults with SZ, HC, and FDR at 7T. It also compared metabolite differences in adults with SZ earlier in the illness (<5 years) compared to later in the illness (>5 years). The results add to the growing body of literature of glutamatergic and GABAergic differences in the ACC in SZ, and report 7T metabolite differences in the CSO, DLPFC, thalamus, and hippocampus, regions implicated in the pathophysiology of SZ. The ACC and the CSO emerged as key regions for metabolite differences that were related to psychopathology and function in SZ in this study. In terms of metabolic abnormalities, the longer illness group was more severely affected than the short illness group for all brain regions.

This study found lower ACC Glu and GABA in the SZ group and higher ACC lactate in the longer illness group, which are consistent with neuroimaging, post-mortem, and behavioral research implicating ACC abnormalities in SZ (7, 21, 29, 63–67). With respect to 7T MRS studies focused on ACC Glu, results have been mixed. Recent 7T MRS work by Posporelis et al. (16), Brandt et al. (15), Kumar et al. (18), and Taylor et al. (36) observed no differences in ACC Glu when comparing either recent onset SZ or chronic SZ with healthy controls. In contrast, Reid et al. (7) showed ACC Glu was lower in first episode patients (within 2 years of starting treatment) than in healthy controls in the dorsal ACC, and these results were similar to those by Wang et al. (19) showing lower Glu in the ACC in first episode patients (2 years from first psychotic symptoms) compared to healthy controls. The current results are similar to these studies in that lower ACC Glu was observed in the SZ group as a whole, and especially so in the longer illness duration group. ACC Glu levels may have functional relevance in schizophrenia, as lower levels were related to poorer functional capacity, functioning, and greater negative symptoms. These results suggest that ACC Glu may be impacted by illness chronicity and contribute to impaired function and negative symptom severity as indicated by findings in the longer illness group. It is not clear as to the cause of lower ACC Glu in schizophrenia since the MRS Glu signal provides a total tissue level and cannot differentiate between glutamate levels in specific cell types (i.e., neurons and glia), location (i.e., intracellular, extracellular, or synaptic), or function (i.e., neurotransmission, GABA synthesis, glutathione synthesis, and protein synthesis). It is possible that lower ACC Glu reflects reduced number or size of glutamatergic pyramidal neurons, consistent with previous research (68), reduced Glu synthesis, and/or impaired Glu-Gln neurotransmitter cycling (69). Interventions to increase ACC Glu levels, especially later in the illness, may reduce negative symptoms and enhance everyday function.

There was a trend for lower ACC GABA in SZ compared to HC which was related to lower level of function as a whole, and greater negative symptom severity in the longer illness group. Three previous 7T MRS studies that did not report differences between SZ and HC (7, 15, 19) but differences in voxel placement, voxel size, acquisition parameters, and illness duration of the SZ group may contribute to this inconsistency. Lower GABA in SZ likely reflects reduced GABA synthesis, consistent with studies of lower GAD67 in SZ (70), resulting in reduced inhibition as suggested from studies of SZ (71).

Our previous paper showed higher ACC lactate in the SZ group compared to the HC group (8). Here, using an overlapping sample but larger N, we did not observe a significant group difference in the ACC when examining adults with SZ, HC, and FDR; however, ACC lactate levels were trend level higher in the longer illness group. This was consistent with the finding of higher CSO lactate in the longer illness group. In the longer illness group, higher ACC lactate was related to greater negative symptoms. Recent work has suggested that lactate may be altered in schizophrenia, potentially due to a bioenergtic or mitochondrial dysfunction (72, 73). The specificity of this relationship to the chronic phase of SZ (>5 years illness duration), in addition to a recent study that did not observe peripheral mitochondrial dysfunction in a clinical high risk for psychosis group (74), suggests that increased lactate may be a long-term consequence of impaired mitochondrial function. However, longitudinal studies are needed to assess the progression of brain lactate levels over the illness course. Further, while adults in both illness duration groups were predominantly treated with antipsychotics, long term antipsychotic medication exposure in rats does not result in increased frontal cortex lactate (72).

The CSO white matter region also revealed higher Gln/Glu ratio and Gln in SZ, which were related to poorer function across all groups, and greater negative symptom severity (Gln/Glu) and greater psychopathology (Gln) in the SZ group. In addition, CSO Glu was lower in the longer illness group and GABA lower in the FDR group. To our knowledge, there is one other study that has examined the CSO at 7T, and no differences in CSO Glu, Gln, or GABA were observed in first episode patients compared to controls (19). Since similar sequences, voxel placement, and voxel size were used, the significant Gln/Glu and Gln findings in this study may be attributed to the longer illness duration of the SZ group. These findings also lend further support to significant white matter alterations in SZ (39, 75) and the possibility that white matter alterations are related to abnormal glutamatergic function (38). Similar to the ACC, the reason for lower Glu or increased Gln/Glu and Gln in SZ is unclear. Abnormalities in the synthesis, degradation, or Glu-Gln cycling could be explanations. Also, specific neural cell types could be affected such as oligodendrocytes in white matter that play an important role in removing synaptic glutamate and the subsequent conversion to glutamine. It should also be noted that glutamine is synthesized in the brain from glutamate and ammonia; ammonia in blood crosses the blood-brain barrier, and if elevated (e.g., due to liver dysfunction or other factors) is known to drive brain glutamine synthesis (76). Blood ammonia levels were not measured in this study, so it is unknown if this is a significant factor or not in the subjects reported here.

There were no significant differences in metabolites across the three groups for the DLPFC, hippocampus, or thalamus. A trend for higher DLPFC Gln in both SZ and FDR compared to HC was found but this was not related to cognitive or functioning measures. Lower DLPFC Glu, lower hippocampal GABA, and higher thalamic mI were found in the longer compared to the shorter illness group. Only thalamus mI was related to a clinical measure, with greater thalamic mI related to more severe general psychopathology. One 7T MRS study of DLPFC did not find any group differences in first episode patients (19), which is in line with these results showing only a trend Gln difference and that lower Glu is likely associated again with illness duration. To our knowledge, there are no other studies examining the hippocampus at 7T in SZ. Finally, there were no group differences for the metabolites of interest in this study in bilateral thalamus, which was similar to another study (19). Another study at 7T that examined metabolite differences the left thalamus from an early illness SZ cohort and HC found higher Gln in the SZ group compared to the HC group (36). Our results showed higher Gln in the SZ group compared to the HC group for the thalamus, but the difference did not reach statistical significance (*p* = 0.064).

The FDR group was not distinguishable except for lower CSO GABA compared to HC and SZ and a similar trend as SZ for higher DLPFC Gln compared to HC. Since the FDR sample size is small, these analyses were restricted to non-parametric tests, and the results should be interpreted with caution. One prior study has compared patients with schizophrenia with both healthy siblings and unrelated control subjects using 7T MRS (17); that study found that reduced GABA in occipitalcortex was specific to the patient group only, but that glutamate was reduced in patients and relatives compared to healthy controls. These results suggest that glutamatergic metabolism may be altered both in patients with schizophrenia as well as those who may have genetic risk for schizophrenia.

There are a few limitations that are worth noting. First, the sample size for the FDR group was small compared to the SZ and HC groups. Second, the macromolecule background present in all short TE MRS studies was handled using the built-in capabilities of LCModel. While this methodology has been used previously and shown to be an effective means of handling the background, it remains unclear whether the proteins and lipids that make up the macromolecule spectrum are different in SZ or FDR. It should also be noted that spectral quality (including factors such as linewidth and SNR) vary depending on location within the human brain; this depends on both the size and location of the MRS voxel. For instance, it is well-known that structures such as the hippocampus are somewhat less favorable compared to more superior and/or posterior brain regions, both because of its small size and also proximity to intracranial air spaces that cause magnetic field inhomogeneity. Lower spectral quality implies more variance in the determination of metabolite concentration values, hence it may be more difficult to demonstrate statistically significant findings for regions with lower spectral quality; in the current study, quality metrics were lower for hippocampus and thalamus as compared to the other three, more superior, brain regions.

This study contributes to the building evidence of *in-vivo* glutamatergic and GABAergic abnormalities and relation to functional and clinical symptoms in schizophrenia. The results emphasize the potential worsening with illness chronicity and indicate the need for future studies focused on the chronic phase of the illness. The study also highlights the importance of lactate and potentially bioenergetic dysfunction in the pathophysiology of SZ, which are likely linked to abnormalities in glutamatergic and GABAergic systems and illness features.

## Data Availability Statement

Data are not readily available because anonymized data sharing was not in the consent form for this study. Requests to access the datasets should be directed to awijtenburg@som.umaryland.edu.

## Ethics Statement

The studies involving human participants were reviewed and approved by University of Maryland Baltimore and Johns Hopkins University School of Medicine Institutional Review Boards. The patients/participants provided their written informed consent to participate in this study.

## Author Contributions

LR and PB had full access to all the data in the study and take responsibility for the integrity of the data and the accuracy of the data analysis, concept and design, obtained funding, and supervision. SW, LR, and PB: drafting of the manuscript. SC: statistical analysis. SW, MW, SK, LR, and PB: administrative, technical, or material support. All authors: acquisition, analysis, or interpretation of data, and critical revision of the manuscript for important intellectual content.

## Conflict of Interest

LR received consulting fees from Otsuka America Pharmaceutical, Inc for educational purposes only for the platform PsychU. The remaining authors declare that the research was conducted in the absence of any commercial or financial relationships that could be construed as a potential conflict of interest.
