# Supplementary material for: Metabolite Alterations in Adults With Schizophrenia, First Degree Relatives, and Healthy Controls: A Multi-Region 7T MRS Study
Source: Front Psychiatry. 2021 May 19;12:656459. doi: 10.3389/fpsyt.2021.656459 (PMC8170030; doi:10.3389/fpsyt.2021.656459)
Supplement: Supplementary file 1 [file Data_Sheet_1.docx]

**Supplement**

*MRS Voxel Composition and Quality Assessments for SZ, HC, and first degree relatives*

In the ACC, there were no differences between groups for proportion of GM (p=0.88), WM (p=0.85), and CSF (p=0.65) as well as FWHM (p=0.76) and SNR (p=0.51) as reported by LCModel. In the CSO, there were no differences between groups for proportion of GM (p=0.49), WM (p=0.48), and CSF (p=0.30) as well as FWHM (p=0.25) and SNR (p= 0.19) as reported by LCModel. In DLPFC, there were no differences between groups for proportion of GM (p=0.54), WM (p=0.55), and CSF (p=0.20) as well as FWHM (p=0.39) and SNR (p=0.46) as reported by LCModel. In the hippocampus, there were no differences between groups for proportion of GM (p=0.74), WM (p=0.65), and CSF (p=0.41) as well as FWHM (p=0.70) and SNR (p=0.21) as reported by LCModel. In the thalamus, there were no differences between groups for proportion of WM (p=0.11), CSF (p=0.26), FWHM (p=0.30), and SNR (p=0.18). There were differences in percentages of GM (p=0.025) such that adults with SZ had lower GM and lower SNR than the HC and FDR groups.

After combining all three groups together, SNR and FWHM differences between the five MRS regions were assessed. An omnibus Friedman test revealed a significant difference between regions for SNR (χ^2^=257, p<0.001) and FWHM (χ^2^=227.6, p<0.001). For SNR, post-hoc Wilcoxon signed rank tests were all significant such that SNR from each region was significantly different from all other regions (range of Z = -5.9 to -8.1, all p’s <0.001). Given that the number of averages was identical for all 5 regions, the differences in voxel size may account for some of these SNR differences between regions. Each brain region was selected because it is believed to play a role in pathophysiology of SZ so the voxel sizes needed to be adjusted to accommodate each specific brain region. Overall, the larger voxel sizes had the highest SNR. For FWHM, post-hoc Wilcoxon signed rank tests were significant for the following comparisons: ACC FWHM-CSO FWHM, ACC_FWHM-DLFPC FWHM, ACC FWHM-hippocampus FWHM, ACC FWHM-thalamus FWHM, CSO FWHM-hippocampus FWHM, CSO FWHM-thalamus FWHM, hippocampus FWHM-thalamus FWHM (range of Z=-5.2 to -7.9, all p’s 0.001) except for DLPFC FWHM and CSO FWHM (Z=-0.03, p=0.978). The FWHM quantified by LCModel is greatly influenced by the achievable shim in each brain region. In order to acquire data from specific brain regions relevant to the study of SZ, some MRS voxels had to be placed in close proximity to the sinuses such as the hippocampus or contained iron deposits such as the thalamus, both of which may negatively impact the shim quality.

*MRS Voxel Composition and Quality Assessments for Short and Long Illness Duration Groups*

In the ACC, there were significant differences between groups for percentage of GM (p=0.049) and trend level differences for WM percentage (p=0.067), FWHM (p=0.068), and SNR (p=0.064). GM percentages were higher, WM percentages were lower, FWHM were narrower, and SNR were higher in short illness duration group versus the long illness duration group. There were no differences in CSF percentage (p=0.87).

In the CSO, there were no differences between groups for percentages of GM (p=0.13), WM (p=0.11), FWHM (p=0.37), and SNR (p=0.13). There was a trend level difference in CSF percentage (p=0.091) such that short illness duration group had less CSF than the longer illness duration group.

In DLPFC, there were no differences between groups for percentages of GM (p=0.14), WM (p=0.38), and CSF (p=0.48). FWHM (p=0.005) and SNR (p=0.001) were significantly different between groups such that the short illness duration group had narrower FWHM and higher SNR than the long illness duration group.

In the hippocampus, there were no differences between groups for percentages of GM (p=0.33), WM (p=0.44), and CSF (p=0.48) as well as FWHM (p=0.93) and SNR (p=0.77).

In the thalamus, there were no differences between groups for percentages of GM (p=0.74), WM (p=0.47), CSF (p=0.41), and FWHM (p=0.41). There was a significant difference for SNR (p=0.013) such that the short illness duration group had higher SNR than the long illness duration group.

*Group Differences between Short Illness Duration SZ Participants and HC*

To assess the effect of age, group metabolite differences (GABA, Glu, Gln, Gln/Glu, and Lac) were assessed between the short illness duration SZ group (N=19) and an age-matched HC subgroup (N=19) using non-parametric tests (Mann-Whitney). In the ACC, Glu was trend level significant between the two groups (U=114, p=0.083) such that Glu was higher in HC than in the short illness duration SZ group. There were no significant differences in the other metabolites. In the CSO, Gln, Gln/Glu, and Lac were significantly different between groups (U=74, p=0.009; U=76, p=0.011; U=21, p=0.037). Gln and Gln/Glu were higher in the short illness duration SZ group compared to HC while Lac was higher in the HC compared to the short illness duration group. There were two trend level differences between groups for Gln (U=100, p=0.051) and Gln/Glu (U=105, p=0.073) in the thalamus. There were no significant metabolite differences in the DLFPC and hippocampus.

*Impact of Age on Metabolite Levels Between Groups*

To assess the impact of age on metabolite levels between the three groups, non-parametric ANCOVAs were conducted for the main metabolites of interest (GABA, Glu, Gln, Gln/Glu, and Lac) in each region with significance set to p<0.05. In the ACC, there were significant group differences for GABA (F(2,83)=3.372, p=0.039) and Glu (F(2,84)=4.078, p=0.02). In the CSO, GABA (F(2,85)=3.587, p=0.032), Gln (F(2,82)=3.398,p=0.038), and Gln/Glu (F(2,82)=4.025,p=0.022) were significant between groups. In the DLFPC, Gln (F(2,82)=2.84, p=0.064) differences between groups remained at trend level. These findings mirror the results highlighted in Table S1 showing that the significant and trend level findings are not a function of age.

To assess the impact of age on the metabolite levels between short and long illness duration groups, non-parametric ANCOVAs were also conducted for all main metabolites with significance set to p<0.05. There were no significant metabolite difference between groups after controlling for age in the ACC (p’s = 0,532-0.977), CSO (p’s = 0.388-0.741), DLPFC (p’s = 0.530-0.940), hippocampus (p’s = 0.376 – 0.685), and thalamus (p’s = 0.332-0.797).

| **Table S1. Metabolite of Interest Levels from 5 Regions for the 3 Groups (in I.U.)** | | | | | |  |
| --- | --- | --- | --- | --- | --- | --- |
|  | SZ (mean ± stdev) | SZ N | FDR (mean ± stdev) | FDR N | HC (mean ± stdev) | HC N |
| Anterior Cingulate |  |  |  |  |  |  |
| GABA* | 1.79 ± 0.25 | 39 | 1.93 ± 0.19 | 11 | 1.87 ± 0.27 | 36 |
| Glu** | 8.81 ± 0.85 | 39 | 9.29 ± 0.70 | 11 | 9.13 ± 0.95 | 37 |
| Gln | 2.06 ± 0.41 | 39 | 1.97 ± 0.33 | 11 | 1.97 ± 0.41 | 37 |
| Gln/Glu | 0.23 ± 0.043 | 39 | 0.21 ± 0.034 | 11 | 0.22 ± 0.045 | 37 |
| Lac | 0.66 ± 0.16 | 35 | 0.60 ± 0.16 | 11 | 0.60 ± 0.11 | 34 |
| Left CSO |  |  |  |  |  |  |
| GABA** | 1.83 ± 0.29 | 40 | 1.64 ± 0.16 | 10 | 1.82 ± 0.22 | 38 |
| Glu | 6.14 ± 0.71 | 40 | 6.01 ± 0.45 | 10 | 6.10 ± 0.48 | 38 |
| Gln** | 1.26 ± 0.27 | 39 | 1.21 ± 0.32 | 10 | 1.10 ± 0.23 | 36 |
| Gln/Glu** | 0.21 ± 0.044 | 39 | 0.20 ± 0.040 | 10 | 0.18 ± 0.037 | 36 |
| Lac | 0.79 ± 0.38 | 22 | 0.58 ± 0.042 | 5 | 0.66 ± 0.20 | 24 |
| Left DLPFC |  |  |  |  |  |  |
| GABA | 1.71 ± 0.30 | 37 | 1.64 ± 0.27 | 10 | 1.72 ± 0.34 | 37 |
| Glu | 7.48 ± 0.078 | 38 | 7.59 ± 0.65 | 11 | 7.35 ± 0.83 | 38 |
| Gln* | 1.59 ± 0.40 | 38 | 1.59 ± 0.44 | 11 | 1.40 ± 0.35 | 36 |
| Gln/Glu | 0.21 ± 0.056 | 38 | 0.21 ± 0.056 | 11 | 0.19 ± 0.049 | 36 |
| Left Hippocampus |  |  |  |  |  |  |
| GABA | 2.17 ± 0.49 | 27 | 2.39 ± 0.54 | 9 | 2.39 ± 0.46 | 28 |
| Glu | 6.77 ± 0.87 | 34 | 6.74 ± 1.15 | 10 | 7.03 ± 0.84 | 31 |
| Gln | 1.80 ± 0.50 | 29 | 1.72 ± 0.55 | 8 | 1.72 ± 0.60 | 27 |
| Gln/Glu | 0.26 ± 0.066 | 29 | 0.24 ± 0.047 | 8 | 0.24 ± 0.074 | 27 |
| Bilateral Thalamus |  |  |  |  |  |  |
| GABA | 2.54 ± 0.55 | 40 | 1.72 ± 0.55 | 11 | 2.50 ± 0.40 | 38 |
| Glu | 6.89 ± 0.94 | 40 | 6.85 ± 0.44 | 11 | 6.94 ± 0.68 | 38 |
| Gln | 1.91 ± 0.64 | 38 | 1.67 ± 0.33 | 11 | 1.63 ± 0.34 | 38 |
| Gln/Glu | 0.27 ± 0.078 | 38 | 0.24 ± 0.041 | 11 | 0.24 ± 0.053 | 36 |
| **p<0.05, *p<0.1, CSO - Centrum Semiovale, DLPFC - Dorsolateral Prefrontal Cortex, FDR - first degree relative, GABA - ɣ-aminobutyric acid, Gln - glutamine, Glu - glutamate, HC - healthy control, Lac - lactate, stdev - standard deviation, SZ – schizophrenia | | | | | | |

| **Table S2. MRS Voxel Composition and Quality Assessments for SZ, HC, and First Degree Relatives as well as Short and Long Illness Duration Groups** | | | | | | |
| --- | --- | --- | --- | --- | --- | --- |
| Region | Group | %GM | %WM | %CSF | FWHM (ppm) | SNR |
| Anterior Cingulate | HC | 0.71 ± 0.037 | 0.18 ± 0.032 | 0.11 ± 0.033 | 0.031 ± 0.006 | 51.7 ± 8.2 |
|  | FDR | 0.71 ± 0.038 | 0.18 ± 0.036 | 0.12 ± 0.038 | 0.031 ± 0.008 | 52.6 ± 9.6 |
|  | SZ | 0.71 ± 0.039 | 0.18 ± 0.040 | 0.11 ± 0.041 | 0.032 ± 0.007 | 50.4 ± 6.9 |
|  | short ill. dur. SZ | 0.73 ± 0.030** | 0.17 ± 0.046 | 0.10 ± 0.034 | 0.030 ± 0.006 | 52.4 ± 5.4 |
|  | long ill. dur. SZ | 0.70 ± 0.042** | 0.19 ± 0.033 | 0.11 ± 0.047 | 0.034 ± 0.007 | 48.7 ± 7.7 |
| Left CSO | HC | 0.12 ± 0.042 | 0.87 ± 0.046 | 0.01 ± 0.006 | 0.035 ± 0.004 | 41.7 ± 7.6 |
|  | FDR | 0.14 ± 0.047 | 0.85 ± 0.054 | 0.012 ± 0.009 | 0.035 ± 0.004 | 42.1 ± 6.1 |
|  | SZ | 0.13 ± 0.043 | 0.86 ± 0.051 | 0.014 ± 0.009 | 0.037 ± 0.006 | 38.3 ± 8.8 |
|  | short ill. dur. SZ | 0.12 ± 0.047 | 0.87 ± 0.054 | 0.011 ± 0.009 | 0.037 ± 0.007 | 40.3 ± 7.7 |
|  | long ill. dur. SZ | 0.14 ± 0.038 | 0.85 ± 0.045 | 0.015 ± 0.009 | 0.038 ± 0.005 | 36.5 ± 9.6 |
| Left DLPFC | HC | 0.42 ± 0.056 | 0.53 ± 0.072 | 0.047 ± 0.025 | 0.036 ± 0.007 | 32.4 ± 7.7 |
|  | FDR | 0.44 ± 0.086 | 0.50 ± 0.12 | 0.060 ± 0.038 | 0.034 ± 0.004 | 33.1 ± 9.6 |
|  | SZ | 0.43 ± 0.054 | 0.52 ± 0.061 | 0.042 ± 0.022 | 0.037 ± 0.005 | 31.3 ± 7.3 |
|  | short ill. dur. SZ | 0.44 ± 0.045 | 0.52 ± 0.061 | 0.038 ± 0.018 | 0.034 ± 0.005** | 35.2 ± 5.3** |
|  | long ill. dur. SZ | 0.42 ± 0.044 | 0.53 ± 0.061 | 0.045 ± 0.025 | 0.039 ± 0.005** | 27.5 ± 6.9** |
| Left Hippocampus | HC | 0.56 ± 0.064 | 0.40 ± 0.067 | 0.036 ± 0.01 | 0.063 ± 0.017 | 12.2 ± 3.8 |
|  | FDR | 0.57 ± 0.072 | 0.40 ± 0.068 | 0.034 ± 0.018 | 0.063 ± 0.022 | 12.5 ± 4.1 |
|  | SZ | 0.57 ± 0.064 | 0.38 ± 0.067 | 0.038 ± 0.013 | 0.060 ± 0.014 | 11.0 ± 2.7 |
|  | short ill. dur. SZ | 0.59 ± 0.075 | 0.37 ± 0.076 | 0.036 ± 0.008 | 0.061 ± 0.018 | 11.3 ± 3.3 |
|  | long ill. dur. SZ | 0.57 ± 0.051 | 0.39 ± 0.058 | 0.040 ± 0.016 | 0.059 ± 0.011 | 10.7 ± 2.0 |
| Bilateral Thalamus | HC | 0.30 ± 0.043** | 0.64 ± 0.049 | 0.06 ± 0.026 | 0.049 ± 0.009 | 18.2 ± 5.7 |
|  | FDR | 0.31 ± 0.021** | 0.64 ± 0.033 | 0.048± 0.019 | 0.045 ± 0.008 | 18.5 ± 5.9 |
|  | SZ | 0.28 ± 0.043** | 0.66 ± 0.052 | 0.053 ± 0.03 | 0.051 ± 0.011 | 15.9 ± 4.7 |
|  | short ill. dur. SZ | 0.28 ± 0.043 | 0.67 ± 0.046 | 0.046 ± 0.014 | 0.049 ± 0.011 | 17.7 ± 4.6** |
|  | long ill. dur. SZ | 0.28 ± 0.043 | 0.66 ± 0.058 | 0.06 ± 0.039 | 0.052 ± 0.011 | 14.2 ± 4.3** |
| **p<0.05, %CSF - percent cerebrospinal fluid, %GM - percent gray matter, %WM - percent white matter, CSO - Centrum Semiovale, DLPFC - Dorsolateral Prefrontal Cortex, FDR - first degree relative, FWHM - full width half maximum, HC - healthy controls, ill dur. – illness duration, SNR - signal to noise ratio, stdev - standard deviation, SZ - schizophrenia | | | | | | |

| **Table S3. Metabolite of Interest Levels from 5 Regions for Short and Long Illness Duration Groups (in I.U.)** | | | |  |
| --- | --- | --- | --- | --- |
|  | Short (mean ± stdev) | Short N | Long (mean ± stdev) | Long N |
| Anterior Cingulate |  |  |  |  |
| GABA | 1.86 ± 0.22 | 18 | 1.73 ± 0.25 | 21 |
| Glu** | 9.23 ± 0.79 | 18 | 8.44 ± 0.73 | 21 |
| Gln | 2.15 ± 0.34 | 18 | 1.98 ± 0.46 | 21 |
| Gln/Glu | 0.23 ± 0.032 | 18 | 0.23 ± 0.052 | 17 |
| Lac* | 0.61 ± 0.13 | 18 | 0.72 ± 0.18 | 21 |
| Left CSO |  |  |  |  |
| GABA | 1.83 ± 0.18 | 19 | 1.82 ± 0.36 | 21 |
| Glu* | 6.28 ± 0.56 | 19 | 6.02 ± 0.82 | 21 |
| Gln | 1.25 ± 0.22 | 18 | 1.27 ± 0.30 | 21 |
| Gln/Glu | 0.20 ± 0.051 | 18 | 0.21 ± 0.038 | 21 |
| Lac** | 0.55 ± 0.12 | 8 | 0.94 ± 0.41 | 14 |
| Left DLPFC |  |  |  |  |
| GABA | 1.72 ± 0.29 | 18 | 1.71 ± 0.32 | 19 |
| Glu** | 7.89 ± 0.52 | 18 | 7.11 ± 0.80 | 20 |
| Gln | 1.65 ± 0.49 | 18 | 1.54 ± 0.32 | 20 |
| Gln/Glu | 0.21 ± 0.070 | 18 | 0.22 ± 0.043 | 20 |
| Left Hippocampus |  |  |  |  |
| GABA* | 2.39 ± 0.48 | 11 | 2.03 ± 0.45 | 16 |
| Glu | 6.66 ± 1.07 | 16 | 6.87 ± 0.66 | 18 |
| Gln | 1.70 ± 0.37 | 13 | 1.87 ± 0.59 | 16 |
| Gln/Glu | 0.25 ± 0.066 | 13 | 0.27 ± 0.068 | 16 |
| Bilateral Thalamus |  |  |  |  |
| GABA | 2.51 ± 0.60 | 19 | 2.56 ± 0.52 | 21 |
| Glu | 6.94 ± 1.00 | 19 | 6.85 ± 0.91 | 21 |
| Gln | 1.83 ± 0.44 | 17 | 1.98 ± 0.76 | 21 |
| Gln/Glu | 0.25 ± 0.055 | 17 | 0.29 ± 0.092 | 21 |
| **p<0.05, *p<0.1, CSO - Centrum Semiovale, DLPFC - Dorsolateral Prefrontal Cortex, FDR - first degree relative, GABA - ɣ-aminobutyric acid, Gln - glutamine, Glu - glutamate, HC - healthy control, Lac - lactate, stdev - standard deviation, SZ - schizophrenia | | | | |

| **Table S4. Fullwidth Half Maximum and SNR from All Three Groups Combined** | | |
| --- | --- | --- |
| Region | FWHM (ppm) ± stdev | SNR ± stdev |
| Anterior Cingulate | 0.032 ± 0.006^a,b,c,d^ | 51.2 ± 7.8^a,b,c,d^ |
| Left Centrum Semiovale | 0.036 ± 0.005^c,d,e^ | 40.2 ± 8.1^b,c,d,e^ |
| Left Dorsolateral Prefrontal Cortex | 0.036 ± 0.006^c,d,e^ | 31.9 ± 7.7^a,c,d,e^ |
| Left Hippocampus | 0.062 ± 0.016^a,b,d,e^ | 11.7 ± 3.4^a,b,d,e^ |
| Bilateral Thalamus | 0.049 ± 0.010^a,b,c,e^ | 17.2 ± 5.4^a,b,c,e^ |
| ^a^significantly different from CSO, ^b^significantly different from DLPFC, ^c^significantly different from hippocampus, ^d^significantly different from thalamus, ^e^significantly different from ACC, ACC – anterior cingulate cortex, CSO – centrum semiovale, DLFPC – dorsolateral prefrontal cortex, FWHM - full width half maximum, SNR - signal to noise ratio, stdev - standard deviation | | |

| **Table S5. Quantifiable Metabolite Levels from 5 Regions for the 3 Groups (in I.U.)** | | | | | |  |
| --- | --- | --- | --- | --- | --- | --- |
|  | SZ (mean ± stdev) | SZ N | FDR (mean ± stdev) | FDR N | HC (mean ± stdev) | HC N |
| Anterior Cingulate |  |  |  |  |  |  |
| Cr | 4.19 ± 0.43 | 39 | 4.30 ± 0.45 | 11 | 4.13 ± 0.39 | 37 |
| GPC | 1.41 ± 0.20 | 39 | 1.42 ± 0.21 | 11 | 1.34 ± 0.21 | 36 |
| GSH | 1.73 ± 0.18 | 39 | 1.71 ± 0.17 | 11 | 1.73 ± 0.14 | 36 |
| mI | 5.77 ± 0.70 | 39 | 5.89 ± 0.61 | 11 | 5.77 ± 0.46 | 37 |
| NAA | 8.21 ± 0.59 | 39 | 8.30 ± 0.62 | 11 | 8.34 ± 0.77 | 37 |
| NAAG | 0.71 ± 0.17 | 36 | 0.68 ± 0.16 | 9 | 0.77 ± 0.18 | 31 |
| PCr | 2.57 ± 0.32 | 38 | 2.62 ± 0.24 | 11 | 2.58 ± 0.31 | 36 |
| Ser | 0.95 ± 0.33 | 29 | 1.03 ± 0.26 | 9 | 1.00 ± 0.38 | 31 |
| Tau | 1.41 ± 0.29 | 39 | 1.31 ± 0.24 | 11 | 1.38 ± 0.25 | 36 |
| tCho | 1.71 ± 0.23 | 39 | 1.69 ± 0.14 | 11 | 1.65 ± 0.20 | 37 |
| tNAA | 8.88 ± 0.61 | 39 | 8.92 ± 0.60 | 11 | 9.04 ± 0.94 | 37 |
| tCr | 6.74 ± 0.37 | 39 | 6.92 ± 0.56 | 11 | 6.65 ± 0.56 | 37 |
| Glx | 10.87 ± 1.09 | 39 | 11.27 ± 0.89 | 11 | 11.10 ± 1.17 | 37 |
| mI+Gly | 6.07 ± 0.63 | 39 | 6.19 ± 0.61 | 11 | 6.16 ± 0.52 | 37 |
| Left CSO |  |  |  |  |  |  |
| Cr | 4.11 ± 0.86 | 40 | 4.02 ± 0.60 | 10 | 3.92 ± 0.43 | 38 |
| GPC | 1.52 ± 0.27 | 40 | 1.52 ± 0.24 | 10 | 1.41 ± 0.27 | 38 |
| GSH | 1.53 ± 0.26 | 40 | 1.49 ± 0.24 | 10 | 1.44 ± 0.21 | 38 |
| mI | 5.75 ± 1.2 | 40 | 5.85 ± 0.82 | 10 | 5.62 ± 0.46 | 38 |
| NAA | 8.37 ± 0.80 | 40 | 8.39 ± 0.76 | 10 | 8.36 ± 0.59 | 38 |
| NAAG | 2.58 ± 0.44 | 40 | 2.55 ± 0.59 | 10 | 2.78 ± 0.41 | 38 |
| PCr | 2.26 ± 0.44 | 36 | 2.23 ± 0.40 | 10 | 2.19 ± 0.30 | 35 |
| tCho | 1.65 ± 0.26 | 40 | 1.63 ± 0.15 | 10 | 1.61 ± 0.22 | 38 |
| tNAA | 10.95 ± 0.91 | 40 | 10.94 ± 1.14 | 10 | 11.14 ± 0.81 | 38 |
| tCr | 6.26 ± 0.70 | 40 | 6.26 ± 0.37 | 10 | 6.05 ± 0.39 | 38 |
| Glx | 7.39 ± 0.84 | 40 | 7.23 ± 0.74 | 10 | 7.18 ± 0.60 | 38 |
| mI+Gly | 5.88 ± 1.2 | 40 | 5.93 ± 0.72 | 10 | 5.77 ± 0.59 | 38 |
| Left DLPFC |  |  |  |  |  |  |
| Cr | 4.06 ± 0.92 | 38 | 4.19 ± 0.43 | 11 | 4.08 ± 0.58 | 38 |
| GPC | 1.37 ± 0.23 | 35 | 1.29 ± 0.21 | 11 | 1.29 ± 0.21 | 36 |
| GSH | 1.66 ± 0.23 | 38 | 1.61 ± 0.28 | 11 | 1.69 ± 0.29 | 38 |
| mI | 5.49 ± 0.91 | 38 | 5.56 ± 0.76 | 11 | 5.46 ± 0.57 | 38 |
| NAA | 8.65 ± 0.70 | 38 | 8.71 ± 0.88 | 11 | 8.88 ± 1.04 | 38 |
| NAAG | 1.26 ± 0.38 | 36 | 1.25 ± 0.26 | 10 | 1.39 ± 0.41 | 34 |
| PCr | 2.23 ± 0.45 | 33 | 2.21 ± 0.32 | 10 | 2.19 ± 0.44 | 32 |
| PE | 0.44 ± 0.54 | 36 | 0.90 ± 0.69 | 11 | 0.58 ± 0.67 | 37 |
| tCho | 1.47 ± 0.23 | 38 | 1.32 ± 0.22 | 11 | 1.39 ± 0.21 | 38 |
| tNAA | 9.86 ± 0.75 | 38 | 9.91 ± 0.96 | 11 | 10.19 ± 1.0 | 38 |
| tCr | 6.10 ± 0.61 | 38 | 6.32 ± 0.58 | 11 | 6.14 ± 0.52 | 38 |
| Glx | 9.07 ± 0.92 | 38 | 9.18 ± 0.89 | 11 | 8.69 ± 1.04 | 38 |
| mI+Gly | 5.88 ± 0.85 | 38 | 5.94 ± 0.72 | 11 | 5.87 ± 0.51 | 38 |

| Left Hippocampus |  |  |  |  |  |  |
| --- | --- | --- | --- | --- | --- | --- |
| mI | 6.89 ± 1.47 | 33 | 6.64 ± 1.71 | 10 | 6.69 ± 1.08 | 31 |
| NAA | 6.32 ± 0.95 | 34 | 5.99 ± 1.11 | 10 | 6.67 ± 0.81 | 31 |
| tCho | 2.0 ± 0.25 | 33 | 1.94 ± 0.27 | 10 | 2.03 ± 0.28 | 31 |
| tNAA | 7.78 ± 1.09 | 34 | 7.99 ± 0.61 | 10 | 8.19 ± 0.94 | 31 |
| tCr | 6.30 ± 0.85 | 34 | 6.12 ± 1.26 | 10 | 6.14 ± 0.73 | 31 |
| Glx | 8.45 ± 1.23 | 34 | 8.33 ± 1.65 | 10 | 8.67 ± 1.21 | 31 |
| mI+Gly | 7.12 ± 1.46 | 33 | 7.02 ± 1.32 | 10 | 7.08 ± 1.05 | 31 |
| Bilateral Thalamus |  |  |  |  |  |  |
| Cr | 5.17 ± 1.26 | 40 | 5.05 ± 0.83 | 11 | 5.18 ± 1.06 | 38 |
| GPC | 1.92 ± 0.35 | 34 | 1.74 ± 0.19 | 11 | 1.83 ± 0.28 | 38 |
| GSH | 1.59 ± 0.37 | 37 | 1.45 ± 0.22 | 11 | 1.55 ± 0.30 | 38 |
| mI | 5.23 ± 1.23 | 39 | 5.25 ± 0.82 | 11 | 5.25 ± 1.33 | 38 |
| NAA | 7.42 ± 0.89 | 40 | 7.36 ± 0.54 | 11 | 7.76 ± 0.72 | 38 |
| NAAG | 1.71 ± 0.64 | 38 | 1.72 ± 0.59 | 10 | 1.65 ± 0.51 | 35 |
| tCho | 1.90 ± 0.34 | 39 | 1.80 ± 0.14 | 11 | 1.88 ± 0.20 | 38 |
| tNAA | 9.09 ± 1.09 | 40 | 8.99 ± 0.47 | 11 | 9.34 ± 0.80 | 38 |
| tCr | 6.13 ± 0.81 | 40 | 6.21 ± 0.38 | 11 | 6.28 ± 0.63 | 38 |
| Glx | 8.76 ± 1.42 | 40 | 8.52 ± 0.67 | 11 | 8.57 ± 0.79 | 38 |
| mI+Gly | 5.44 ± 1.16 | 39 | 5.33 ± 0.77 | 11 | 5.35 ± 1.22 | 38 |
| **p<0.05, Cr - creatine, CSO - Centrum Semiovale, DLFPC - Dorsolateral Prefrontal Cortex, FDR - first degree relative, Glx - glutamate+glutamine, GPC - glycerophosphocholine, GSH - glutathione, Gly - glycine, HC - healthy control, Lac - lactate, mI - myo-Inositol, NAA - N-acetylasparate, NAAG - N-acetylaspartylglutamate, SZ - schizophreniastdev - standard deviation, tCho - total choline, tCr - total creatine, tNAA - total N-acetylaspartate | | | | | | |

| **Table S6. Quantifiable Metabolite Levels from 5 Regions for Short and Long Illness Duration Groups (I.U.)** | | | | |
| --- | --- | --- | --- | --- |
|  | Short (mean ± stdev) | SZ N | Long (mean ± stdev) | HC N |
| Anterior Cingulate |  |  |  |  |
| Cr | 4.13 ± 0.31 | 18 | 4.25 ± 0.51 | 21 |
| GPC | 1.39 ± 0.19 | 18 | 1.42 ± 0.20 | 21 |
| GSH | 1.73 ± 0.17 | 18 | 1.72 ± 0.19 | 21 |
| mI | 5.70 ± 0.51 | 18 | 5.84 ± 0.83 | 21 |
| NAA | 8.44 ± 0.53 | 18 | 8.01 ± 0.58 | 21 |
| NAAG | 0.74 ± 0.15 | 16 | 0.68 ± 0.18 | 20 |
| PCr | 2.60 ± 0.27 | 18 | 2.54 ± 0.36 | 20 |
| Tau | 1.41 ± 0.28 | 18 | 1.41 ± 0.31 | 21 |
| tCho | 1.64 ± 0.21 | 18 | 1.77 ± 0.23 | 21 |
| tNAA | 9.14 ± 0.53 | 18 | 8.66 ± 0.60 | 21 |
| tCr | 6.73 ± 0.29 | 18 | 6.74 ± 0.43 | 21 |
| Glx** | 11.4 ± 1.00 | 18 | 10.4 ± 0.99 | 21 |
| mI+Gly | 6.02 ± 0.51 | 18 | 6.11 ± 0.73 | 21 |
| Left CSO |  |  |  |  |
| Cr | 3.98 ± 0.65 | 19 | 4.23 ± 1.02 | 21 |
| GPC | 1.54 ± 0.24 | 19 | 1.50 ± 0.30 | 21 |
| GSH | 1.46 ± 0.21 | 19 | 1.59 ± 0.29 | 21 |
| mI | 5.33 ± 0.67 | 19 | 6.13 ± 1.43 | 21 |
| NAA | 8.67 ± 0.77 | 19 | 8.10 ± 0.74 | 21 |
| NAAG | 2.53 ± 0.36 | 19 | 2.62 ± 0.50 | 21 |
| PCr | 2.07 ± 0.33 | 17 | 2.42 ± 0.47 | 19 |
| tCho | 1.56 ± 0.22 | 19 | 1.73 ± 0.27 | 21 |
| tNAA | 11.2 ± 0.88 | 19 | 10.7 ± 0.90 | 21 |
| tCr | 5.99 ± 0.41 | 19 | 6.50 ± 0.82 | 21 |
| Glx | 7.49 ± 0.55 | 19 | 7.29 ± 1.05 | 21 |
| mI+Gly | 5.49 ± 0.67 | 19 | 6.23 ± 1.45 | 21 |
| Left DLPFC |  |  |  |  |
| Cr | 4.05 ± 0.60 | 18 | 4.08 ± 1.15 | 20 |
| GPC | 1.31 ± 0.21 | 18 | 1.43 ± 0.24 | 17 |
| GSH | 1.67 ± 0.22 | 18 | 1.66 ± 0.23 | 20 |
| mI | 5.22 ± 0.56 | 18 | 5.74 ± 1.10 | 20 |
| NAA | 8.78 ± 0.76 | 18 | 8.53 ± 0.63 | 20 |
| NAAG | 1.27 ± 0.39 | 17 | 1.26 ± 0.37 | 19 |
| PCr | 2.10 ± 0.35 | 16 | 2.36 ± 0.52 | 17 |
| PE | 0.63 ± 0.54 | 18 | 0.26 ± 0.49 | 18 |
| tCho | 1.39 ± 0.16 | 18 | 1.55 ± 0.26 | 20 |
| tNAA | 10.0 ± 0.82 | 18 | 9.72 ± 0.68 | 20 |
| tCr | 5.99 ± 0.44 | 18 | 6.18 ± 0.74 | 20 |
| Glx** | 9.54 ± 0.63 | 18 | 8.65 ± 0.96 | 20 |
| mI+Gly | 5.66 ± 0.58 | 18 | 6.07 ± 1.0 | 20 |

| Left Hippocampus |  |  |  |  |
| --- | --- | --- | --- | --- |
| mI | 6.93 ± 1.78 | 33 | 6.81 ± 1.15 | 17 |
| NAA | 6.32 ± 0.79 | 34 | 6.31 ± 1.10 | 18 |
| tCho | 1.98 ± 0.32 | 33 | 2.02 ± 0.18 | 18 |
| tNAA | 7.66 ± 1.22 | 34 | 7.89 ± 0.98 | 18 |
| tCr | 6.24 ± 1.07 | 34 | 6.35 ± 0.62 | 18 |
| Glx | 8.22 ± 1.28 | 34 | 8.65 ± 1.18 | 18 |
| mI+Gly | 7.01 ± 1.71 | 33 | 7.23 ± 1.22 | 18 |
| Bilateral Thalamus |  |  |  |  |
| Cr | 4.86 ± 0.96 | 19 | 5.46 ± 1.45 | 21 |
| GPC | 1.85 ± 0.27 | 16 | 1.97 ± 0.41 | 18 |
| GSH | 1.56 ± 0.35 | 18 | 1.62 ± 0.39 | 19 |
| mI** | 4.68 ± 1.06 | 18 | 5.71 ± 1.19 | 21 |
| NAA | 7.58 ± 1.03 | 19 | 7.28 ± 0.74 | 21 |
| NAAG | 1.58 ± 0.49 | 18 | 1.83 ± 0.75 | 20 |
| tCho | 1.85 ± 0.26 | 18 | 1.95 ± 0.40 | 21 |
| tNAA | 9.14 ± 1.26 | 19 | 9.05 ± 0.93 | 21 |
| tCr | 5.84 ± 0.83 | 19 | 6.39 ± 0.70 | 21 |
| Glx | 8.68 ± 1.40 | 19 | 8.83 ± 1.47 | 21 |
| mI+Gly** | 4.87 ± 1.02 | 18 | 5.93 ± 1.07 | 21 |
| **p<0.05, Cr - creatine, CSO - Centrum Semiovale, DLFPC - Dorsolateral Prefrontal Cortex, FDR - first degree relative, Glx - glutamate+glutamine, GPC - glycerophosphocholine, GSH - glutathione, Gly - glycine, HC - healthy control, Lac - lactate, mI - myo-Inositol, NAA - N-acetylasparate, NAAG - N-acetylaspartylglutamate, SZ - schizophreniastdev - standard deviation, tCho - total choline, tCr - total creatine, tNAA - total N-acetylaspartate | | | | |

| **Table S7. Metabolite of Interest Levels from 5 Regions for Short Illness Duration and Age-Matched Healthy Controls (in I.U.)** | | | | |
| --- | --- | --- | --- | --- |
|  | Short (mean ± stdev) | Short N | HC (mean ± stdev) | HC N |
| Anterior Cingulate |  |  |  |  |
| GABA | 1.86 ± 0.22 | 18 | 1.96 ± 0.20 | 19 |
| Glu* | 9.23 ± 0.79 | 18 | 9.55 ± 0.53 | 19 |
| Gln | 2.15 ± 0.34 | 18 | 2.10 ± 0.34 | 19 |
| Gln/Glu | 0.23 ± 0.032 | 18 | 0.22 ± 0.036 | 19 |
| Lac | 0.61 ± 0.13 | 18 | 0.57 ± 0.10 | 18 |
| Left CSO |  |  |  |  |
| GABA | 1.83 ± 0.18 | 19 | 1.82 ± 0.22 | 19 |
| Glu | 6.28 ± 0.56 | 19 | 6.17 ± 0.51 | 19 |
| Gln** | 1.25 ± 0.22 | 18 | 1.04 ± 0.22 | 17 |
| Gln/Glu** | 0.20 ± 0.051 | 18 | 0.17 ± 0.027 | 17 |
| Lac** | 0.55 ± 0.12 | 8 | 0.72 ± 0.22 | 12 |
| Left DLPFC |  |  |  |  |
| GABA | 1.72 ± 0.29 | 18 | 1.86 ± 0.33 | 19 |
| Glu | 7.89 ± 0.52 | 18 | 7.69 ± 0.72 | 19 |
| Gln | 1.65 ± 0.49 | 18 | 1.44 ± 0.37 | 18 |
| Gln/Glu | 0.21 ± 0.070 | 18 | 0.19 ± 0.047 | 18 |
| Left Hippocampus |  |  |  |  |
| GABA | 2.39 ± 0.48 | 11 | 2.41 ± 0.48 | 12 |
| Glu | 6.66 ± 1.07 | 16 | 7.13 ± 0.92 | 13 |
| Gln | 1.70 ± 0.37 | 13 | 1.57 ± 0.51 | 13 |
| Gln/Glu | 0.25 ± 0.066 | 13 | 0.22 ± 0.050 | 13 |
| Bilateral Thalamus |  |  |  |  |
| GABA | 2.51 ± 0.60 | 19 | 2.48 ± 0.39 | 19 |
| Glu | 6.94 ± 1.00 | 19 | 7.00 ± 0.56 | 19 |
| Gln* | 1.83 ± 0.44 | 17 | 1.56 ± 0.32 | 19 |
| Gln/Glu* | 0.25 ± 0.055 | 17 | 0.22 ± 0.043 | 19 |
| *p<0.05, **p<0.1, CSO - Centrum Semiovale, DLPFC - Dorsolateral Prefrontal Cortex, FDR - first degree relative, GABA - ɣ-aminobutyric acid, Gln - glutamine, Glu - glutamate, HC - healthy control, Lac - lactate, stdev - standard deviation, SZ - schizophrenia | | | | |
